# Mycelium-Based Composites in Art, Architecture, and Interior Design: A Review

**DOI:** 10.3390/polym14010145

**Published:** 2021-12-31

**Authors:** Maciej Sydor, Agata Bonenberg, Beata Doczekalska, Grzegorz Cofta

**Affiliations:** 1 Department of Woodworking and Fundamentals of Machine Design, Faculty of Forestry and Wood Technology, Poznań University of Life Sciences, 60-637 Poznań, Poland; 2 Institute of Interior Design and Industrial Design, Faculty of Architecture, Poznan University of Technology, 60-965 Poznań, Poland; agata.bonenberg@put.poznan.pl; 3 Department of Chemical Wood Technology, Faculty of Forestry and Wood Technology, Poznań University of Life Sciences, 60-637 Poznań, Poland; beata.doczekalska@up.poznan.pl (B.D.); grzegorz.cofta@up.poznan.pl (G.C.)

**Keywords:** biomaterials, bio-composites, bio design, mycelium-based composites, biopolymers, interior design, architecture, wood, mycelium, fungi, patent documents

## Abstract

Mycelium-based composites (MBCs) have attracted growing attention due to their role in the development of eco-design methods. We concurrently analysed scientific publications, patent documents, and results of our own feasibility studies to identify the current design issues and technologies used. A literature inquiry in scientific and patent databases (WoS, Scopus, The Lens, Google Patents) pointed to 92 scientific publications and 212 patent documents. As a part of our own technological experiments, we have created several prototype products used in architectural interior design. Following the synthesis, these sources of knowledge can be concluded: 1. MBCs are inexpensive in production, ecological, and offer a high artistic value. Their weaknesses are insufficient load capacity, unfavourable water affinity, and unknown reliability. 2. The scientific literature shows that the material parameters of MBCs can be adjusted to certain needs, but there are almost infinite combinations: properties of the input biomaterials, characteristics of the fungi species, and possible parameters during the growth and subsequent processing of the MBCs. 3. The patent documents show the need for development: an effective method to increase the density and the search for technologies to obtain a more homogeneous internal structure of the composite material. 4. Our own experiments with the production of various everyday objects indicate that some disadvantages of MBCs can be considered advantages. Such an unexpected advantage is the interesting surface texture resulting from the natural inhomogeneity of the internal structure of MBCs, which can be controlled to some extent.

## 1. Introduction

Fungi can use many types of by-products as substrates for growth. When mycelium penetrates a substrate, it acts as a natural self-assembling binder, holding a loose mixture in a monolithic form, creating a solid composite of biopolymers cellulose matrix and very dense chitin reinforcement. Mycelium can fill the volume with a very dense network; one gram of soil can contain up to 600 km of hyphae [1]. The mycelium growth pattern is related to the availability of food resources, water and environmental conditions, which constantly modify the network topology. The adaptive behaviour of fungi allows them to cope with various ephemeral resources, competition, damage, and predation in a completely different manner from multicellular plants or animals [2]. In nature, the organic matter for fungal growth comes from the remains of plant and animal organisms and their metabolites. In industrial conditions, various types of biological post-consumer wastes and by-products such as wood, straws, husks, chaws, and bagasse can be used as substrates for mycelial growth [3].

Mycelium-based composites are used in construction, packaging, and in the production of various types of products. MBCs are also well suited to applied arts. Philip Ross is the author of the “Hy-Fi” tower-pavilion presented at the “MoMA’s PS1” exhibition in 2014 [4], in this building structure he combined wooden beams with MBC, thus compensating for the low mechanical strength of MBC. The artist is the author of several patent applications and scientific publications in this field [5]. Pascal Leboucq designed “The Growing Pavilion” constructed by Company New Heroes in 2019, a temporary event space at Dutch Design Week constructed with panels grown from mushroom mycelium supported on a timber frame. The Redhouse Architecture Bureau (Cleveland, OH, USA) promotes the use of wood construction waste, such as panels and window frames, which can be defragmented and re-bonded with mycelium and then used to build houses [6]. In Indonesia, Mycotech, Block Research Group and the Karlsruhe Institute of Technology built a prototype spatial structure called “MycoTree” made of various biocomposites, with the addition of sugar cane and cassava root waste (2017) [7]. At Milan Design Week 2019, Carlo Ratti presented an installation entitled “Round Garden”, built from a sequence of arches made of MBCs. The installation fits into the natural context and surroundings [8]. Various artists and designers have designed different mycelium-based products: e.g., Aniela Hoitink 2016 textiles, Erica Klarenbeek 2013 3D printed furniture, Jonas Edvard and Sebastian Cox 2013 lamps, Kristel Peeters and Mycofabrication 2009 shoes. Think tank Terreform ONE and non-profit organization Genspace have developed a series of seating furniture made of Mycoform (2016) [9]. Mycelium is an alternative to wood dust in 3D printing [10]. A group of British architects Blast Studio and Bio-Digital Matter Lab managed to 3D print a column of mycelium-based materials (2018) [4]. Team BioBabes printed 3D MBCs objects using polylactic acid to act as a temporary mycelium scaffold (“Hyper Articulated Myco-Morphs” 2016–2017) [11]. A number of cultural organizations research and popularize MBCs, like Futurium in series of exhibition “Mind the Fungi. Art & Design Residencies” in Berlin since 2019 [12], or Somerset House in series of cultural events “Mushrooms: The Art, Design and Future of Fungi” in London 2020 [13].

Mycelium-based composites have been reported as inventions since at least 2007 and are also the subject of scientific research. Taking into account the great potential and numerous advantages of such a material, it was considered appropriate to review the scientific literature and patent documents supplementing these sources of knowledge with our own experience in the field of manufacturing interior furnishings made of this type of interesting biocomposite. The main aim of the article is to synthesize information from the scientific literature, patent documents, and own experience to identify barriers and possibilities for an effective implementation of mycelium-based composites in industrial manufacturing, especially when applied to decorative objects used in architectural interior design of apartments.

## 2. Results of the Literature Review

**Figure 1:**
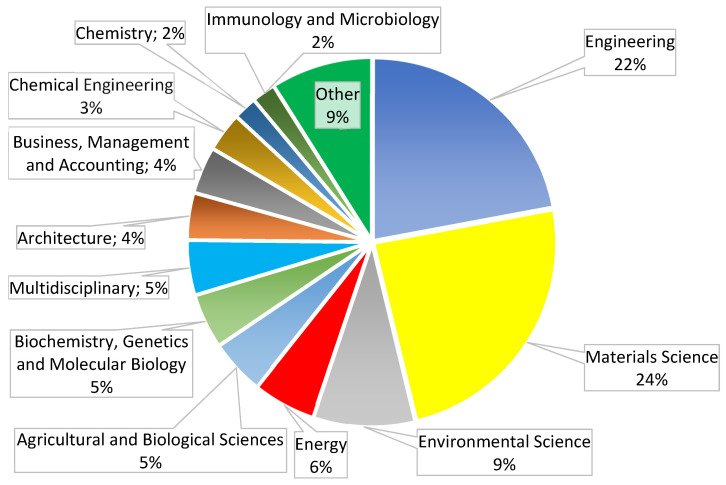
Subject areas of scientific articles on mycelium-based composites.

At least 92 research papers have been published on mycelium-based composites (72 original articles [14,15,16,17,18,19,20,21,22,23,24,25,26,27,28,29,30,31,32,33,34,35,36,37,38,39,40,41,42,43,44,45,46,47,48,49,50,51,52,53,54,55,56,57,58,59,60,61,62,63,64,65,66,67,68,69,70,71,72,73,74,75,76,77,78,79,80,81,82,83,84,85], one being a hybrid of original and review articles [86], and 19 review articles [87,88,89,90,91,92,93,94,95,96,97,98,99,100,101,102,103,104,105]. The oldest article is from 2012 [14], the newest is from November, 2021 [81]. The analyzed articles are assigned to 19 subject areas. The two main research areas are “Materials Science” and “Engineering” (Figure 1).

**Figure 2:**
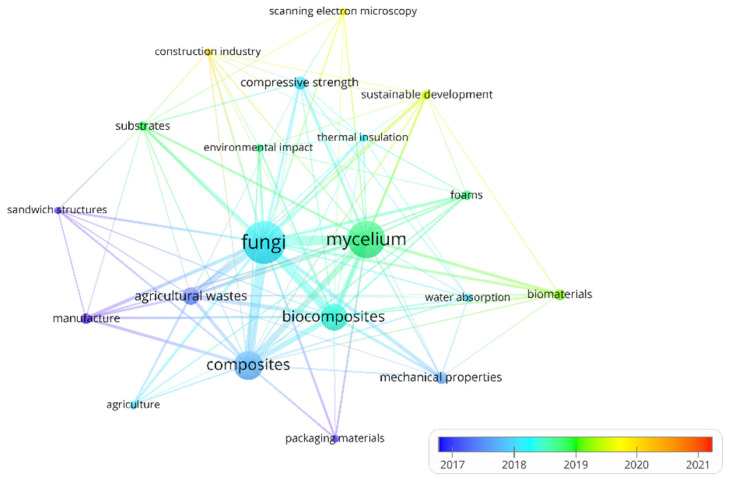
“Author keywords” associations in scientific articles on mycelium-based composites.

Over 130 different “author keywords” are used in the articles. Associations and frequency of co-existence for 20 most frequently used “author keywords” are shown in Figure 2.

In the Figure 2, the frequency of occurrence of the keywords varies with time in color. VOSviewer was used; minor editorial changes have been made in the keywords: singular and plural forms of nouns (“fungus” = “fungi”, “material” = “materials” etc.), the notation (“bio-composites” = “biocomposites” etc.), synonyms (“fungal mycelium” = “mycelium”, “composite materials” = “composites”, “bio-based composites” = “biocomposites” and “manufacturing process” = “manufacture”). Yellow color, turning red, indicates keywords used in the most recent articles. As can be seen, these are the words “sustainable development”, “scanning electron microscopy”, “construction industry” and “agricultural robots”. This shows the changing research interests in this field.

The five most cited articles according to Scopus are summarized in Table 1.

There are different purposes for the research carried out. The vast majority of research focuses on finding out how to properly shape the constructional properties of the material. The objectives and results of selected research works on mycelium-based composites are collected in Table 2.

In 2016–2021, at least 20 scientific review articles were also published. The most important of these articles are listed in Table 3.

## 3. Results of Patents Documents Analysis

A granted patent is an administrative decision: area and time limited, issued by the patent office, it provides protection for a feasible, new, non-obvious and potentially profitable solution. The basis for such a decision is a patent application, which requires an unambiguous description of the essence of the invention. The patent application also provides a priority date, i.e., the date of disclosure of the invention. The priority date can, for example, be the presentation of the invention at a trade fair or the publication of a description of the invention. Most often, however, it is the date when the invention is filed with the patent office. Some patent applications become granted patents. Inventions considered profitable by their owners are filed in many patent jurisdictions around the world. Subsequent applications may differ slightly from their prototypes in terms of content, the differences result mainly from the refinement of descriptions, as well as the rejection of some patent claims by various patent offices. The main patent documents are patent applications and granted patents from many patent offices, they form the so-called patent families. Thus, each patent family describes one invention, the date of its creation is given by its first application.

Patent documents were searched on the basis of the following keywords: mycelium; mycological; fungi; biopolymers; biomaterials; biocomposites. These words were searched for in the “TAC” sections of patent documents (TAC = title OR abstract OR claim). Searches were made in the International Patent Classification areas: C08*, C12N*, B27N* B32B* oraz B32B*, and the list of documents was reviewed, limiting it to issues related to the production of plastics such as foams, boards and blocks used in construction, furniture, the automotive industry, as packaging and as artistic products. Thus, documents dealing with the production of woven fabric, i.e., all non-structural materials used in the manufacturing technique, were omitted. Publicly available databases and analytical tools such as Google Patents, The Lens were used, and the results of queries were exported to MS Excel for further analysis.

**Figure 3:**
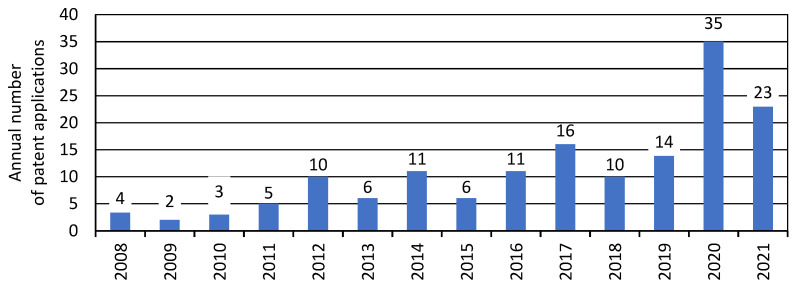
Annual number of patent applications according to publication year.

As a result of the analysis, 212 patent documents were identified: 153 patent applications, 55 patents granted on the basis of some of these applications, and additionally 2 amended applications, 1 amended patent and 1 patent of addition. They constitute 67 extended families, and thus describe 67 different technological and product inventions related to mycelium-based composites. The oldest document was received by the United States Patent and Trademark Office on 12 December 2007 [106], while the last of the analysed documents was on 9 April 2021 [107]. The annual numbers of patent applications, according to the years of their publication, are shown in Figure 3.

Data for 2021 is incomplete, not all patent documents from this year are indexed in databases. The data on the annual number of patents presented in Figure 3 show a significant increase in patent applications in the last two years.

**Figure 4:**
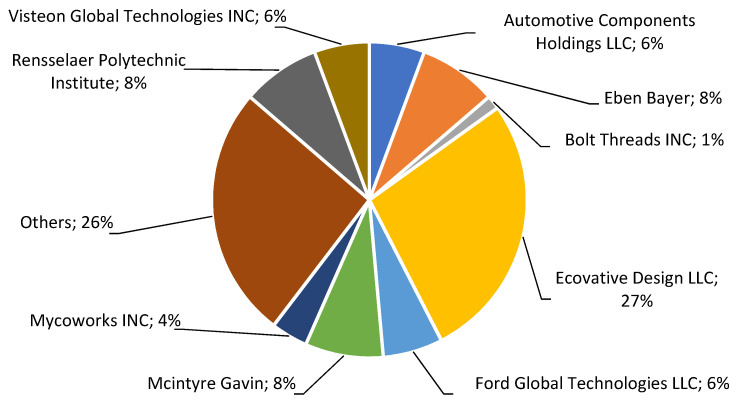
Shares of companies in the total number of patent applications.

There are significant 9 people and organizations among the owners of patent documents. This is shown in Figure 4 as shares in overall number of patent documents.

The owner of the largest number of patent documents is Ecovative Design LCC (Albany, NY, USA), which has 27% of industrial property in this area (58 documents). Other persons and institutions affiliating many documents are: Eben Bayer and Mcintyre Gavin (both related to Ecovative Design LCC) and Rensselaer Polytechnic Institute (Troy, MI, USA) (17 documents each), also Ford Global Technologies LLC and Automotive Components Holdings LLC, both owned by Ford Motor Company headquartered in Dearborn, MI, USA (13 and 12 documents, respectively).

**Figure 5:**
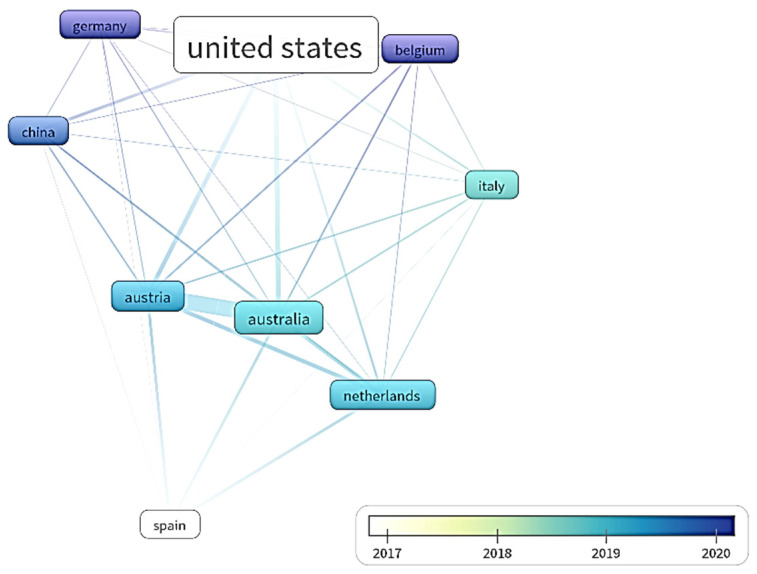
Links between countries in patent documents.

The analysis of patent documents shows 9 main countries related with the mycelium-based composites (Figure 5). The largest number of affiliated patent documents is in the USA. However, the latest documents are affiliated in Germany, Belgium and China.

In 29 patent families there is at least one granted patent, these families are summarized in Table 4, presenting one selected patent from each such patents family.

More than 200 patent documents make it impossible to “intuitively” indicate the key inventions in the field of mycelium-based composites. Undoubtedly, the first patent application (US 2008/0145577 A1, “Method for producing grown materials and products made thereby” [106], filing date 12 December 2007) is important, but there are likely to be other influential inventions in this field. In the 2019 scientific article on the review of wood screw patents [136], the following criteria were proposed for the identification of important patents:

- The size of the patent family—the assumption: “only an invention with high application potential can be submitted for protection in many patent offices because the patent procedure is paid”.
- Number of citations of a patent document in other, later patent documents—the assumption: “if multiple patent documents refer to a particular document, it indicates that this document describes (and perhaps at least partially solves) a significant problem.

Using these two criteria, the most influential patent documents for mycelium-based technology were listed in Table 5.

The generalized MBCs production protocol can be compiled from research articles, patent documents, or open source manuals (e.g., [142]). Such a general protocol includes:

(1) The chosen mycelium specie is pre-grown in a Petri dish with a growth medium solidified with agar.
(2) The substrate for the culture of mycelium is homogenized (the substrate is a mix of selected biopolymers with defined granulation and proportion). The substrate is also sterilized to kill or deactivate all microorganisms in it.
(3) The pre-grown mycelium and sterile water are added to the substrate. Additional nutrients can also be added. The inoculated substrate is packed in a sterile mould (a bag or a container).
(4) The mycelium grows trough the substrate in a controlled micro-climate (temperature, air humidity, without light). The mycelium composite can be created initially in the mould to its internal reinforcement, and then outside the mould to solidify its surface.
(5) The mycelium composite is sterilized to end the growth process and then dried to the target moisture content.
(6) A pressing, machining, coating or other required product post-processing is applied.

The review of patent documents shows that biofoam composites and layered structures with mycelium-based composites can be used in building structures as structural materials (e.g., the core of sandwich panels and gap fillers), interior finishing materials (e.g., wall panels) and floors), as well as materials for portable home furnishings (furniture and other portable items) and packaging materials. They can have an insulating function due to their low heat conductivity or a sound-absorbing function. Biocomposites can therefore be an alternative to synthetic foams found in automotive bumpers, doors, roofs, engine cavities, boot linings, dashboards, and seats because the mycelium-based material has the same or better ability to absorb impacts, insulate, dampen sound and provide lightweight construction in the car from typical synthetic foams. The material also showed good fire resistance. Applications in the construction industry are mainly limited to fire-proof thermal and acoustic insulators. So far, the use of this innovative biocomposite in the construction industry has been limited only to a small scale and to exhibition installations.

Considering all the ecological advantages of mycelial and bio-substrate composites, the question arises, why such materials are not used very widely. Potential reasons for this may be problems with low mechanical properties, high water absorption, lack of Life Cycle Assessment information for this material, and lack of standard production methods and standardized methods for testing material properties.

## 4. Mycelium-Based Material in Elements of Interior Design—Case Study

Even though the mycelium-based composites is currently studied mostly for purposes in which visual or aesthetical aspects are insignificant, like packaging, experiments performed by the authors suggest that it can be successfully used for creating interior design elements. Mycelium-based materials embrace a new aesthetics characterized by imperfections and irregularities through natural and spontaneous growth, thus achieving a unique structure, as in wood. The physical and geometric properties of objects evolve and change slightly over time. These properties make it an unusual and challenging material. Different textures that characterize the material samples depend on how the substrate has been formed before the growth; the material’s surface has visible natural fibres and dominating natural mycelium colouring: off-whites with yellow or brownish irregularities in more mature areas. The user perceives these characteristics as organic, warm, and natural, which influences the typology of products that could be created.

**Figure 6:**
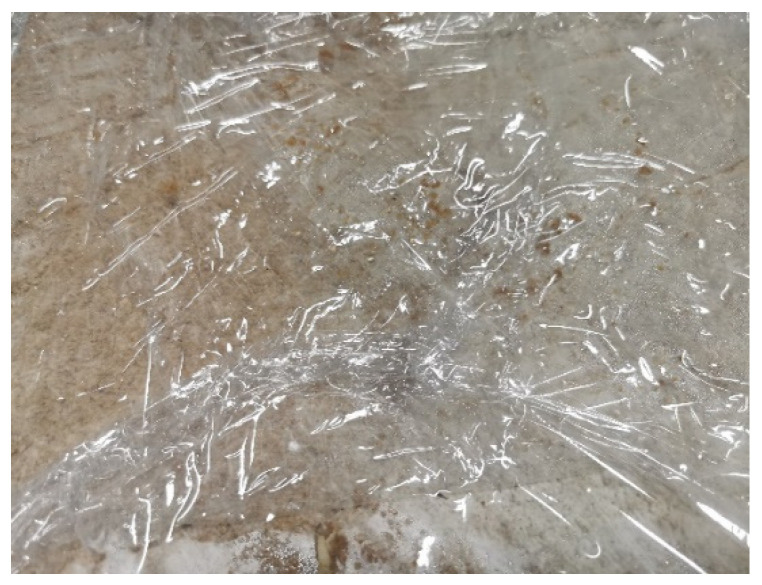
Mycelium-based material during growth (design and photo: A. Bonenberg).

**Figure 7:**
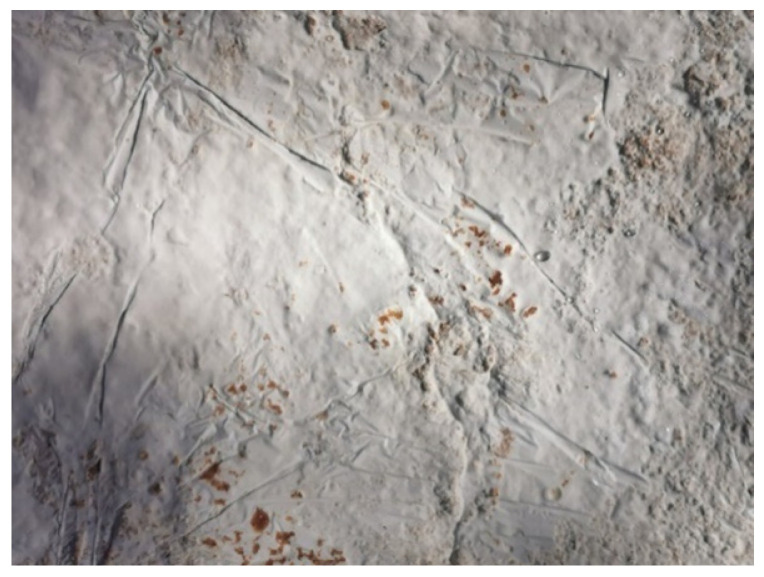
Mycelium-based material after growth: smooth surface with (design and photo: A. Bonenberg).

**Figure 8:**
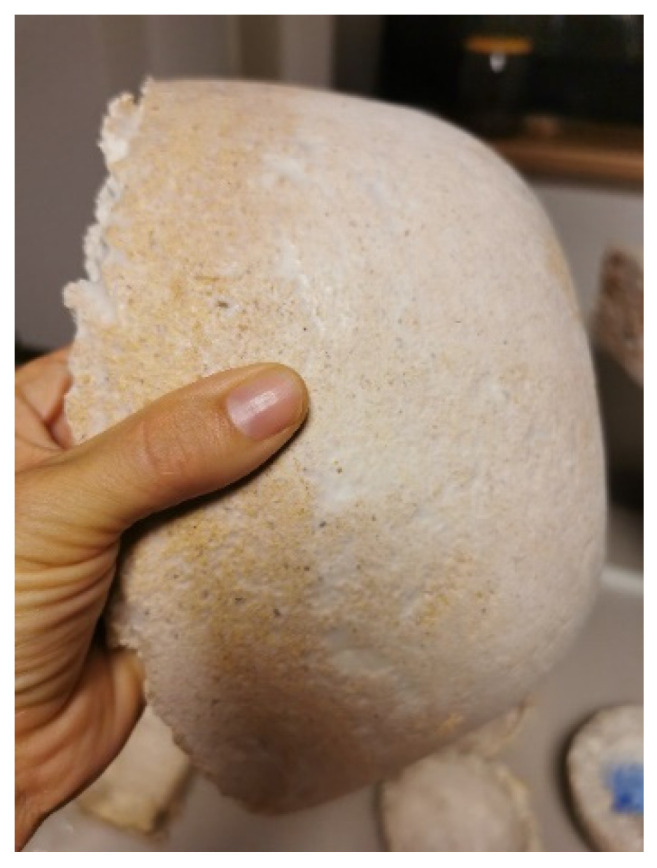
Mycelium-based composite: smooth texture, with visible fibres (design and photo: A. Bonenberg).

**Figure 9:**
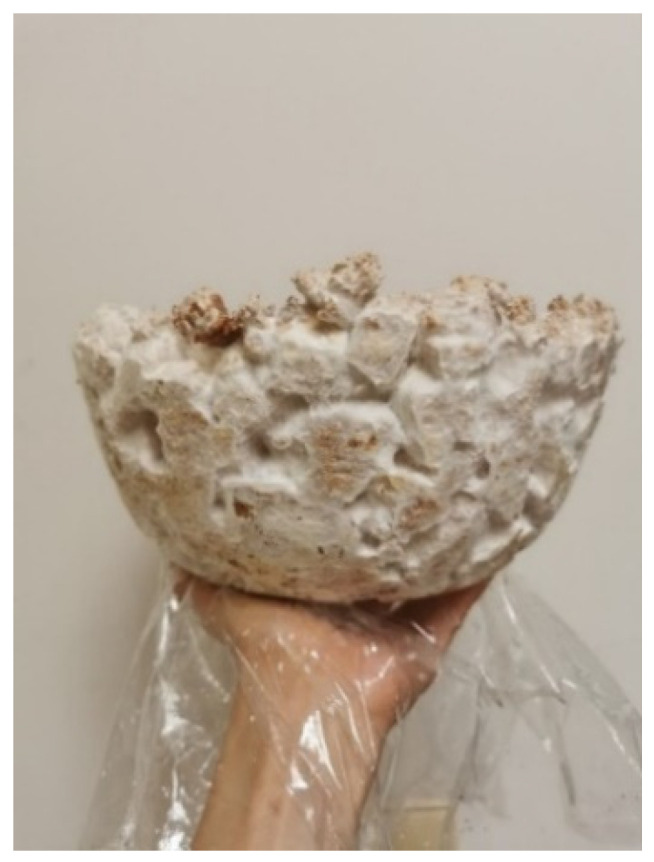
Mycelium-based composite: rough texture, deriving from substrate fragmentation (design and photo: A. Bonenberg).

**Figure 10:**
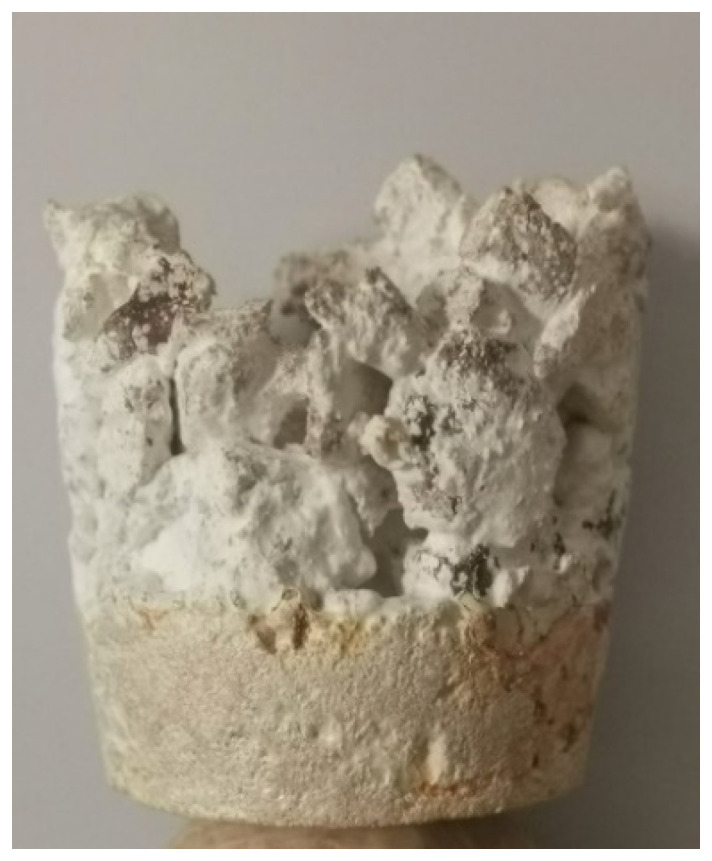
Mycelium-based composite: smooth and rough textures, combined in one object (design and photo: A. Bonenberg).

The first shapes obtained from mycelium-based material by Agata Bonenberg were simple panels that allowed the growth and maturation of the material to be observed (Figure 6 and Figure 7). Then spherical objects were created to study the emergence of different textures: smooth (Figure 8), rough (Figure 9). The object shown in Figure 10 combines both; it has a smooth well-fragmented substrate at the bottom, and an uneven, rough part at the top. This opens interesting possibilities for future projects.

**Figure 11:**
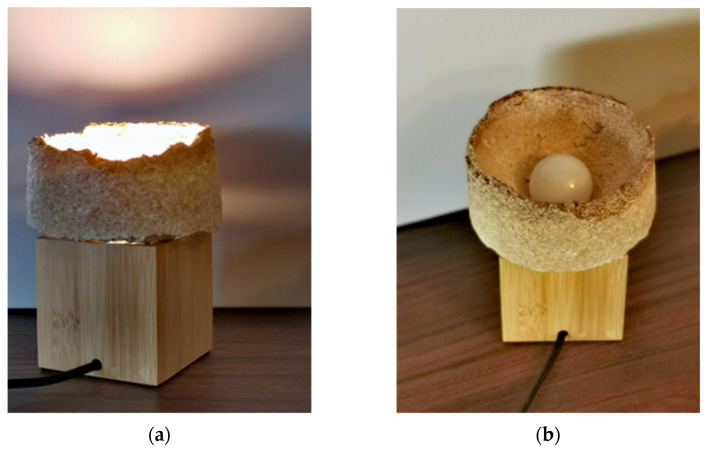
Mycelium-based light fixture: (**a**)—with lamp on, (**b**)—general appearance (design and photo: A. Bonenberg).

**Figure 12:**
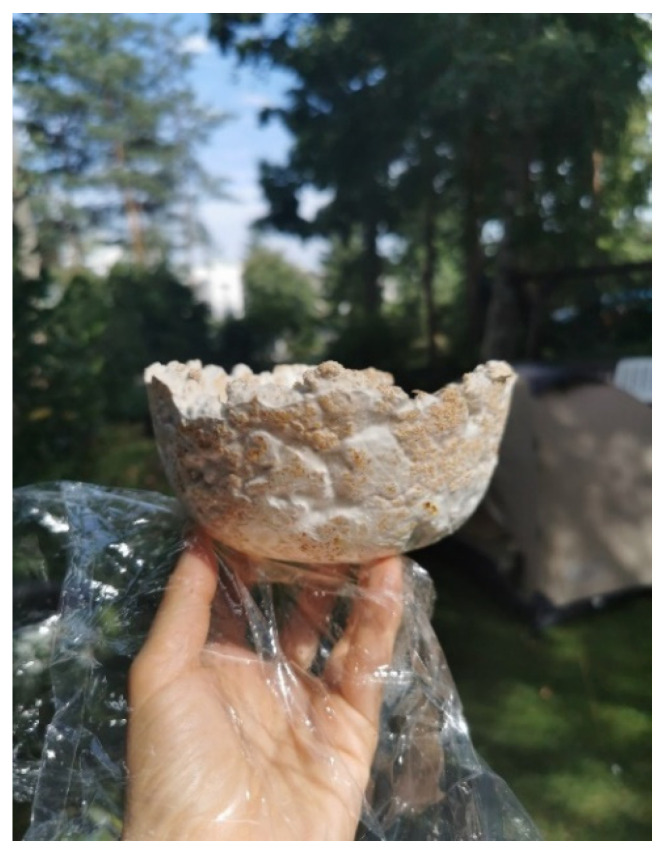
Mycelium-based semi-finished object (design and photo: A. Bonenberg).

Experimentation with textures and shapes of forms has led to preliminary product development and production. The designs of a table light fixture, a table bowl, and a coffee table have been executed. In each of these projects, mycelium-based elements had to be combined with other materials. The author has chosen natural components such as timber to match the design’s pro-ecological spirit and give overall natural “touch”. The small table lamp is a good example of this approach: a mycelium-grown, cylindrical lampshade has been fixed on a simple cubical timber base (Figure 11 and Figure 12).

**Figure 13:**
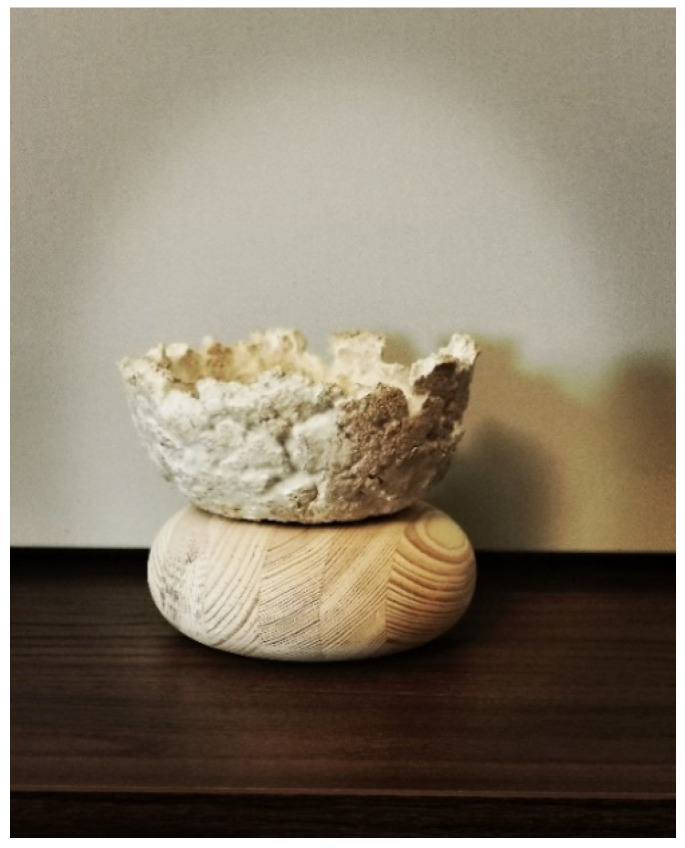
Finished object: mycelium-based bowl fixed to the rough-timber torus-shape base (design and photo: A. Bonenberg).

Similarly, a container bowl was created, where an upper part of the object was fixed to the rough-timber torus-shaped base (Figure 12 and Figure 13). Again, the look of the object is “organic”. At the same time, the heavier wooden base gives the bowl its functional stability.

**Figure 14:**
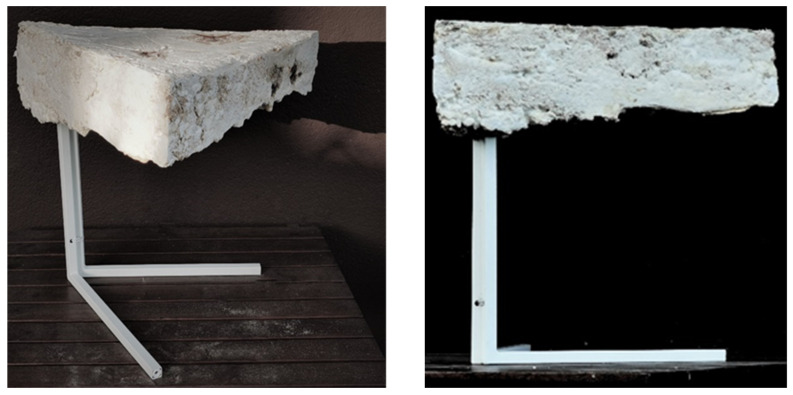
Coffee table with mycelium-based tabletop (design and photo: A. Bonenberg).

Another artifact created is a coffee table where a mycelium-based tabletop has been grown on a metal frame, ensuring its structural stability (Figure 14). The tabletop is thick but light, with well-consolidated smooth surfaces from the top and sides, but an uneven and rough texture can be perceived from the bottom. In addition, there is a clear contrast between the thin steel legs of the table and the thick-bodied top. Finally, the triangular shape gives the object expressive, characteristic looks.

The challenge of unconventional materials is the technique of fastening elements [143,144]. The new material requires a new approach in this field, which will be a further direction of our activities.

## 5. Conclusions

Regarding the current excessive dependence of the construction and production industry on hydrocarbon-containing materials occurring within Earth’s crust; taking into account the abundance of waste and industrial by-products, it is necessary to make greater use of the advantages of biomaterials with a low carbon footprint [145,146,147,148,149,150,151,152]. The following conclusions can be drawn from the reviewed content presented:

1. MBCs (mycelium-based composites) offer favourable production price, ecological value, and high artistic value. Their weaknesses are insufficient design properties and not fully known reliability (quality during use), therefore both scientific research and engineering creativity, which is manifested by patents documents, are heading in this direction.
2. A review of the scientific literature shows that the material parameters of MBCs can be adjusted to the needs: by selecting the type of substrate and fungus species, by controlling the growth conditions, the method of inactivation of the mycelium after growth, and the drying method. In this way, it is possible to meet certain requirements, e.g., increase the structural load-bearing capacity to an acceptable level and reduce the affinity with water, and additionally improve the acoustic and thermal insulation. However, the problem is the almost infinite number of combinations: properties of the input biomaterials, characteristics of the mushroom species, and parameters during growth and subsequent processing of the MBC.
3. The review of patent documents shows that two current technological challenges are related to the creation of MBCs with the properties required by the final product. Especially, looking for an effective method of increasing strength, for example by increasing the density, the search for a method of obtaining a more homogeneous internal structure.
4. The described own technological experiments, consisting of the production of various everyday objects, indicate that some disadvantages of MBCs can be considered advantages. Such an unexpected advantage is the interesting and unrepeatable surface texture resulting from the natural unevenness of the internal structure of MBCs, which can be controlled to some extent.

The presented results of the analysis of a wide variety of literature and own technological experiments suggest that the share of mycelium-based composites in industrial production and construction will increase, despite certain limitations of this innovative class of materials in terms of manufacturing difficulties, insufficient strength, unknown durability and reliability, and challenges in fastening technology. These problems will be gradually solved or at least significantly minimized. This is supported by the fundamental advantages of these types of bio-composites, i.e., the ability to produce from by-products or waste, low energy requirements for production, biodegradability and artistic values.

## Figures and Tables

**Table 1:** The most frequently cited articles on mycelium-based composite materials according to Scopus.

Year	Title	Type	No. of Citations	Reference
2017	Advanced Materials from Fungal Mycelium: Fabrication and Tuning of Physical Properties	original	128	[28]
2017	Morphology and mechanics of fungal mycelium	original	80	[36]
2017	Mycelium composites: A review of engineering characteristics and growth kinetics	review	74	[90]
2012	Fungal mycelium and cotton plant materials in the manufacture of biodegradable moulded packaging material: Evaluation study of select blends of cotton by-products	original	74	[14]
2019	Fabrication factors influencing mechanical, moisture- and water-related properties of mycelium-based composites	original	66	[52]

**Table 2:** Parameters and aims of mycelium-based composites production in scientific research.

Fungi	Substrate	Product/Application	Main Results (MBC = Mycelium-Based Composites)	Reference
*Ganoderma* sp.	Cotton-based (carpel, seed hull) starch, and gypsum	Packaging material	MBC meets or exceeds the characteristics of extruded polystyrene foam	[14]
Not specified (possibly as [14])	Rice straw, hemp seed, kenaf fibre, switch grass, sorghum fibre, cotton bur fibre, flax shive	Insulation panel	Optimal performance at the noise frequency of 1000 Hz. MBC are comparable to polyurethane foam board and are better than plywood	[15]
*G. lucidum, P. ostreatus*	Cellulose and potato-dextrose broth (PDB)	Fibrous mycelium film	The substrate should be homogeneous. The PDB in the substrate increases the stiffness of MBC	[28]
*T. versicolor*	Glass fines, wheat grains, and rice hulls	Fire safe mycelium biocomposites	MBC are safer than the typical construction materials: producing much lower heat release rates, less smoke and CO_2_ and longer time to flashover. Composites with glass fines had the best fire performance	[46]
*T. ochracea, P. ostreatus*	Beech sawdust, rapeseed straw, bran. Non-woven cotton fibre	Board	Straw-based mycelium composites are stiffer and less moisture-resistant than cotton based	[52]
*T. versicolor, P. brumalis*	Wheat straw, rice hulls, sugarcane bagasse, blackstrap molasses, wheat grains, malt extract	Pure mycelium	Mycelium grew slow on rice hull, sugarcane bagasse and wheat straw. Liquid blackstrap molasses accelerates growth, outperforming laboratory malt extracts.	[49]
*T. versicolor*	Flax dust, flax long, wheat straw dust, wheat straw, hemp fibres and pine wood shavings	Thermal insulation	The thermal conductivity and water absorption of MBCs are comparable to those of rock wool, glass wool, and extruded polystyrene. The mechanical properties depend more on the fibre arrangement than on the chemical composition of the fibres	[57]
Not specified (white-rot basidiomycete mycelium)	Mixture of spruce, pine, and fir	Particleboard	Cellulose nanofibers added to the substrate improved the mechanical properties of MBC by 5%	[53]
*P. ostreatus, F. oxysporum*	Sodium silicate	Pure mycelium	3% sodium silicate improve thermal stability. The *P. ostreatus* compared to the *F. oxysporum* beter improve material thermal stability (higher decomposition temperature and residual weight, lower degradation rate)	[59,84]
*G. lucidum, P. ostreatus*	Clay, sawdust, bleached and unbleached cellulose	Printed cylinders	The mycelium improves the 3D printing (better water resistance, material stiffness and surface hardness)	[73]

**Table 3:** List of scientific review publications for mycelium-based composites for art, architecture, and interior design.

Year	Reference	No. of Cited Documents	No. of Citations in Scopus	Main Findings
2016	[88]	32	22	A production cost model is described which includes labour, material and overhead costs for structured sandwich products produced from MBCs.
2017	[90]	170	74	1. MBCs are kind of biopolymer foam, but most studies admit that mechanical performance can be improved in the future. 2. Current use is limited to the packaging and chosen construction applications. New applications have been proposed (acoustic dampers, super absorbents, paper, textiles, structural and electronic parts).
2018	[91]	21	34	1. MBCs can be used for a variety of purposes with the advantage of a lower cost and the better disposal than polystyrene that is an environmental problem. 2. The biggest challenge is the negative public perception of fungus-derived products.
2019	[94]	11	26	MBCs are profitable renewable and degradable material and have the potential to replace petroleum-based materials.
2019	[92]	108	37	Improvement in know-how is expected to improve the mechanical properties and to standardize the productive process, whereas insulation and thermal properties already have shown competitive results.
2020	[86]	58	21	1. There is a correlation between raw input material composition and final material properties. 2. MBCs have implications for sustainable architecture and products. 3. The unique aesthetics of MBCs should be further explored and more clearly identified.
2020	[96]	80	44	1. Fungal biorefinery upcycles by-products into cheap and sustainable composite materials. 2. Can replace foam, timber and plastic insulation, door cores, panels, flooring, furnishings. 3. Low density and thermal conductivity, high acoustic absorption, and fire safety. 4. MBCs are suitable as thermal and acoustic insulation foams.
2021	[98]	77	6	1. MBCs are more suitable for thermal and acoustic insulation than synthetic foam and wood fibres. 2. MBCs are stiff, lightweight and biodegradable, thus are an alternative to petroleum-based packaging materials.
2021	[101]	101	0	The process of engineering affects the properties of MBCs. Bioreactor designs such as tray, packed bed and millilitre reactors, influence of mycelium growth conditions and strategies for controlling mycelium microenvironment are discussed to allow optimal process development.
2021	[102]	118	0	1. MBCs are advantageous as packaging materials with sufficient acoustic, and thermal insulation, slightly worse than expanded polystyrene. 2. The standardized process to produce an optimized material property has yet to be identified, production is less standardized than conventional engineering materials, and it is not clear how to customize the substrates for a particular species of fungi to optimize the composite mechanics.
2021	[103]	80	0	1. MBCs support a circular economy. 2. Finding the ways of enhancing their physicochemical properties will expand the application areas. 3. The properties of MBCs are competitive with those of synthetic polymers used in construction, interior architecture, and other industries.
2021	[104]	94	2	With the wide variety of fungal species and substrates available, MBCs can improve environmental sustainability of many industrial products.

**Table 4:** Granted patents.

Order No.	Patent No., Application Year–Granted Year, Reference	Details
1	US 9,485,917 B2, 2007–2016, [108]	ED (Ecovative Design LLC). Method for producing grown materials and products made thereby
2	US 8,001,719 B2, 2009–2011, [109]	ED. Method for producing rapidly renewable chitinous material using fungal fruiting bodies and product made thereby
3	US 8,313,939 B2, 2010–2012, [110]	FGT, ACH (Ford Global Technologies LLC, Automotive Components Holdings LLC). A method of making a moulded automotive part with a liquid fungal mixture.
4	US 8,298,810 B2, 2010–2012, [111]	
5	US 8,227,233 B2 [112]	
6	US 8,227,224 B2 [113]	FGT, ACH. Method of making moulded part comprising mycelium coupled to mechanical device
7	US 8,227,225 B2 [114]	FGT, ACH. Plasticized mycelium composite and method
8	US 8,283,153 B2 [115]	FGT, ACH. Mycelium structures containing nanocomposite materials and method
9	US 8,298,809 B2 [116]	FGT, ACH. Method of making a hardened elongate structure from mycelium
10	CN 102,329,512 B [117]	Ford Global Technologies LLC. The sheet stock mycelium of cutting and method
11	US 9,410,116 B2, 2011–2016, [118]	Mycoworks Inc. building materials
12	US 9,879,219 B2, 2012–2018, [119]	ED. A method of producing a chitinous polymer derived from fungal growth
13	CA 2,834,095 C, 2012–2018, [120]	ED. Dehydrated mycelium panels.
14	US 10,154,627 B2, 2013–2018, [121]	ED. Growing mycological biomaterials in tools that are consumed or enveloped during the growth process
15	FR 3,006,693 B1 2013–2016, [122]	Menuiseries Elva. A method of producing a composite material based on natural fibres inoculated with mycelium and parts obtained with this method
16	US 9,253,889 B2 2012–2016 [123]	ED. Sheet built-in an electrical circuit
17	US 9,085,763 B2, 2013–2015, [124]	ED. Production dehydrated mycelium elements to form tissue morphology using Pycnoporus cinnabarinus
18	AU 2013/251269 B2, 2013–2015, [125]	ED. Self-supporting composite material
19	US 10,144,149 B2, 2014–2018, [126]	ED. Stiff mycelium bound part and method of producing stiff mycelium bound parts
20	US 9,394,512 B2, 2015–2016, [127]	ED. Method for growing mycological materials
21	US 9,469,838 B2, 2015–2016, [128]	Mycoworks Inc. Set of mycelium-based materials with wood timber
22	CN 105,292,758 B 2016–2017, [129]	Shenzhen Zeqingyuan Technology Dev Service Co Ltd., Univ Sichuan Agricultural. Production method for biomass packing material
23	AU 2015/271912 B2, 2015–2020, [130]	ED. Method of manufacturing a stiff engineered composite
24	US 9,914,906 B2, 2016–2018, [131]	ED. Process for solid-state cultivation of mycelium on a lignocellulose substrate
25	CN 106,148,199 B, 2016–2019, [132]	Jiangxi University of Technology. Agricultural waste-based mycelium material with good a cushion performance and mechanical property
26	CN 106,633,989 B, 2016–2019, [133]	Shenzhen Zeqingyuan Technology Development Service Co Ltd. Using bagasse as fungi-based biomass packaging material of major ingredient and preparation method thereof
27	US 10,604,734 B2, 2017–2020, [134]	University of Alaska Anchorage. Thermal insulation material from mycelium and forestry by-products
28	KR 102,256,335 B1, 2019–2021, [135]	Lee Beom Geun. Eco-friendly packing materials comprising mushroom mycelium and the process for the preparation thereof
29	US 11,015,059 B2, 2019–2021, [107]	Bolt Threads Inc. Composite material, and methods for production thereof

**Table 5:** Most influenced patent documents.

No.	Patent Document	Extended Patent Family Size	Number of Citations of the Patent Document in Other Patent Documents
1	US 2008/0145577 A1 “Method for producing grown materials and products made thereby” [106]	43	44
2	US 2012/0270302 A1 “Method for Making Dehydrated Mycelium Elements and Product Made Thereby” [137]	15	4
3	WO 2019/099474 A1 “Increased Homogeneity of Mycological Biopolymer Grown into Void Space” [138]	12	8
4	US 2012/0135504 A1 “Method for Producing Fungus Structures” [139]	11	20
5	US 2018/0282529 A1 “Solution Based Post-Processing Methods for Mycological Biopolymer Material and Mycological Product Made Thereby” [140]	9	5
6	US 2020/0024577 A1 “Method of Producing a Mycological Product and Product Made Thereby” [141]	7	4

## Data Availability

All data, models, and code generated or used during the study appear in the submitted article.
